# Oropharyngeal obstruction and respiratory system compliance are linked to ventilatory control parameters in pediatric obstructive sleep apnea syndrome

**DOI:** 10.1038/s41598-022-22236-7

**Published:** 2022-10-15

**Authors:** Plamen Bokov, Imene Boujemla, Boris Matrot, Karen Spruyt, Jorge Gallego, Christophe Delclaux

**Affiliations:** 1 Service de Physiologie Pédiatrique-Centre du Sommeil, AP-HP, Hôpital Robert Debré, INSERM NeuroDiderot, Université de Paris-Cité, 48, Boulevard Sérurier, 75019 Paris, France; 2 Service d’Oto-Rhino-Laryngologie, AP-HP, Hôpital Robert Debré, 75019 Paris, France; 3 INSERM NeuroDiderot, 75019 Paris, France

**Keywords:** Physiology, Diseases, Pathogenesis

## Abstract

Instable ventilatory control is an endotypic trait of obstructive sleep apnea syndrome (OSAS). This study aimed to evaluate the relationships between the anatomical compromise of the upper (oro- and naso-pharynx) and lower airways and ventilatory control (measured by chemical loop gain) in otherwise healthy children suffering from moderate to severe OSAS (apnea hypopnea index ≥ 5/hour). The children underwent ear, nose and throat examination, measurement of impedance of the respiratory system that allowed characterizing peripheral lung mechanics using the extended Resistance-Inertance-Compliance model. Physiologically constrained analytical model based on tidal breathing analysis allowed for the computation of steady-state plant gain, steady-state controller gain (CG0) and steady-state loop gain (LG0). Medium-frequency components of the feedback control system were then deduced. Fifty children (median age 11.2 years) were enrolled. Oropharyngeal obstruction was associated with decreased CG0 (0.6 [0.2; 1.0] vs 1.5 [0.5; 6.6] L.s^− 1^.mmHg^− 1^, *p *= 0.038) and LG0 (0.4 [0.2; 1.1] vs 1.2 [0.4; 9.3], *p *= 0.027), while nasal obstruction did not modify ventilatory control parameters. In a multivariate analysis Medium-Frequency PG was negatively related to minute ventilation and respiratory system compliance. Both upper (tonsil hypertrophy) and lower (compliance of respiratory system) airways are linked to ventilatory control in children with moderate to severe OSAS.

## Introduction

Obstructive sleep apnea syndrome (OSAS) affects 1–4% of children^1^ and is associated with significant cardiovascular, behavioral and neurocognitive morbidity. Instability of the control of ventilation is now increasingly recognized as a pathophysiological factor of OSAS, along with factors as anatomically compromised airways, low respiratory threshold and insufficient neuromuscular activation of pharyngeal dilators^2^. Nevertheless, the degree of contribution of ventilatory control abnormalities to the pathogenesis of OSAS in children remains debated^3^. Loop gain (LG), controller gain (CG), and plant gain (PG), which reflect the stability of ventilatory control, chemoreceptor sensitivity and the pulmonary control of blood gas in response to a change in ventilation, respectively, have been evaluated in children with OSAS. While some investigators demonstrated an increase in LG in some adult patients suffering from OSAS^4^, two independent groups demonstrated an increase in PG in children with OSAS while their LG was not different from that of non-OSAS children due to a reduction of CG^5,6^. These two groups established a positive correlation between PG and OSAS severity assessed by the apnea–hypopnea index (AHI), which suggests that high PG may play a more important role in pediatric OSAS. The mechanisms explaining this increase in PG deserve to be studied since it could lead to therapeutic approaches.

PG is related to lung function as by definition it depicts the modification of alveolar CO_2_ after a variation of ventilation. An abnormally elevated PG may result from reduced lung volumes, a reduced ability to eliminate CO_2_ adequately such as in ventilation/perfusion mismatch and decreased pulmonary blood flow^7^. Pulmonary function abnormalities have been associated with OSAS. In obese children, van Eyck and colleagues showed that correlations between sleep-related respiratory parameters (respiratory disturbance index, mean SaO_2_, SaO_2_ nadir) and FEV_1_ remained significant after correction for BMI z-score, demonstrating an independent relationship between OSAS and airflow limitation^8^. In the study of Zerah and colleagues^9^, specific respiratory conductance was linked to OSAS severity and variables that influenced this conductance were distal airway obstruction, morphological upper airway abnormalities and the AHI, highlighting the contribution of both upper and lower airways to adult OSAS. Among upper airways, both nasal and oropharyngeal obstruction can contribute to OSAS^10,11^.

There are also arguments for the contribution of lower and upper airway obstruction to altered ventilatory control. In children with persistent asthma with and without OSAS, the OSAS group was found to have higher PG than the non-OSAS group^12^. As well, the subjects with more severe OSAS and abnormal lung function had higher PG and lower CG relative to the rest of the population studied. The link between upper airway compromise and ventilatory control is important to confirm since Armoni Domany and colleagues demonstrated that six months following adenotonsillectomy, there was a significant decrease in the elevated PG in the OSAS group, while no change observed in the control group^6^. Thus, this study suggested that the mechanisms leading to increased PG are also secondary to upper airway abnormalities.

The objective of our study in otherwise healthy children suffering from moderate to severe OSAS was to assess the relationships between their degree of anatomical compromise of the upper (oro- and naso-pharynx) and lower airways and ventilatory control. A two-compartment lung model allowed assessing the respective contributions of central and peripheral respiratory system. We focused on this group of OSAS since there is a therapeutic indication^10^ and delineation of underlying endotypes deserves to be done.

## Methods

### Participants

Patients were included prospectively as part of our day hospital evaluation for moderate to severe OSAS. The evaluations performed during the hospitalization were: clinical examination with collection of height, weight, neck circumference, oral examination, lung function tests, ear, nose and throat (ENT) examination by an otolaryngologist (IB) together with a measurement of nasal obstruction (acoustic rhinometry) and study of ventilatory control using a recording of tidal ventilation.

The inclusion criteria were symptoms suggestive of OSAS (snoring, apnea, restless sleep, oral breathing) and an AHI ≥ 5/h in otherwise healthy children 3–18 years of age (asthma included) with or without obesity. Non-inclusion criteria were midface deficiency, marked mandibular hypoplasia, prematurity (based on a report of birth at least four weeks earlier), genetic disorders and ongoing treatment for OSAS. This study conformed to the standards set by the latest revision of the *Declaration of Helsinki* and was approved by the Ethics Committee of Robert Debré University Hospital (study registration identity PHENOSAS: N° 2018–416), and the database of collected data was declared to the French regulatory agency (CNIL). Informed consent was obtained from all subjects and their legal guardian(s).

All recordings were performed on the day of the ENT examination during an in-hospital stay, in the morning before the ENT examination. Recordings of tidal breathing were performed as previously described^13^, lasting 20 min, with the first 5 min being discarded. During the recordings, subjects were awake and sitting in a calm and non-stimulating atmosphere. Flow rate, end-tidal PO_2_ (P_ET_O_2_) and end-tidal PCO_2_ (P_ET_CO_2_) were continuously monitored, and signals were digitized using the MP-100 system (Biopac System Inc., Santa Barbara, CA) at a rate of 50 Hz; the data were stored for further analysis.

### End-expiratory CO_2_ slope

The quality of the end-tidal CO_2_ slope is important to take into account when considering its ability to reflect alveolar CO_2_^14^. We proposed a method for the calculation of a corrected expiratory slope (see Supplementary Information). This procedure permitted us to search for outliers corresponding to subjects that could have poorly performed during the tidal breathing recording.

### Loop gain model

We used a constrained bivariate (minute ventilation (${\dot{\mathrm{V}}}_{\mathrm{e}}$) and P_ET_CO_2_) analytical model that allowed us to calculate the components of CG and PG (steady-state gains, time constants of the gains and circulatory delays)^13^.

Briefly, the plant that describes the relationship between ventilation and alveolar PCO_2_ (P_A_CO_2_) is modelled as a first order time delay system with a gain (called steady-state plant gain PG0) and a time constant (τ_p_, which represents the time it takes for a P_A_CO_2_ to reach 63% of its final value after a step change in ventilation).

1 $${\text{PG}}\left( {\text{s}} \right) = \frac{{\Delta {\text{P}}_{{{\text{A}}\;{\text{CO}}_{2} }} \left( {\text{s}} \right)}}{{\Delta {\dot{\text{V}}}_{{\text{e}}} \left( {\text{s}} \right)}} = \frac{{{\text{PG0}}}}{{{1} + {\text{s}}\tau_{{\text{p}}} }}$$

where s denotes the frequency.

The “controller” describes the relationship between P_a_CO_2_ and ventilatory drive that equals ventilation in awake subjects. In this model, the controller is modelled using a static gain (CG0, or steady-state controller gain) and a time constant (τ_c_). Changes in P_A_CO_2_ are delayed by approximately 6 s before reaching the peripheral chemoreceptors. Delays in the time domain are modelled as exponentials in the frequency domain, hence:

2 $${\text{CG}}\left( {\text{s}} \right) = \frac{{\Delta {\dot{\text{V}}}_{{\text{e}}} \left( {\text{s}} \right)}}{{\Delta {\text{P}}_{{{\text{aCO}}_{{2}} }} \left( {\text{s}} \right)}} = \frac{{{\text{CG0}}}}{{{1} + {\text{s}}\tau_{{\text{c}}} }}e^{{ - {\text{sD}}}}$$

where P_a_CO_2_ is the arterial PCO_2_ in the vicinity of the chemoreceptors and D is the delay.

Thus, this model contained only five parameters (PG0, CG0, τ_p_, τ_c_, and D).

By definition, PG0 should be negative and CG0 should be positive; hence, a condition of model fit failure was the observation of non-physiological gain values.

The model was fitted on the changes from baseline (mean) levels of the ventilatory parameters (${\dot{\mathrm{V}}}_{\mathrm{e}}$ and P_ET_CO_2_) that were obtained from tidal breathing measurements while awake, and thus, the analyses were specifically related to chemosensitivity. Since the medium frequency band (corresponding to oscillations of 5–15 breaths/cycle) spans the range of cycle durations of periodic breathing observed experimentally, we focused on this frequency range as was previously done^13^.

### In-laboratory polysomnography

Polysomnography studies were performed overnight. An Alice 6 LDx polysomnography system (Philips, Murrysville, PA) recorded the following parameters: chest and abdominal wall motion using respiratory inductance plethysmography, heart rate by electrocardiogram, arterial oxygen saturation by pulse oximetry, airflow using a 3-pronged thermistor, nasal pressure by a pressure transducer, electroencephalographic leads (C3/A2, C4/A1, F3/A2, F4/A1, O1/A2, O2/A1), left and right electrooculograms, submental electromyogram, and tibial electromyogram. Study participants were also recorded with an infrared video camera. Experienced pediatric sleep physicians scored patients using standard pediatric sleep scoring criteria^15^. The definition of moderate to severe OSAS was sleep disorder breathing symptoms and an apnea hypopnea index (AHI) ≥ 5 episode/h^10^.

### Pulmonary function tests

Impedance of the respiratory system was measured using an impulse oscillatory system (IOS: Master Scope Body, Carefusion Technologies, Yorba Linda, California, USA), as previously described^16^. We used the following IOS variables: resistance and reactance at 5, 10, 15, 20, 25, 30 and 35 Hz. Since OSAS may be an independent risk factor for small airway disease^9^, we used an additional lung model to characterize this small airway disease.

**Figure 1:**
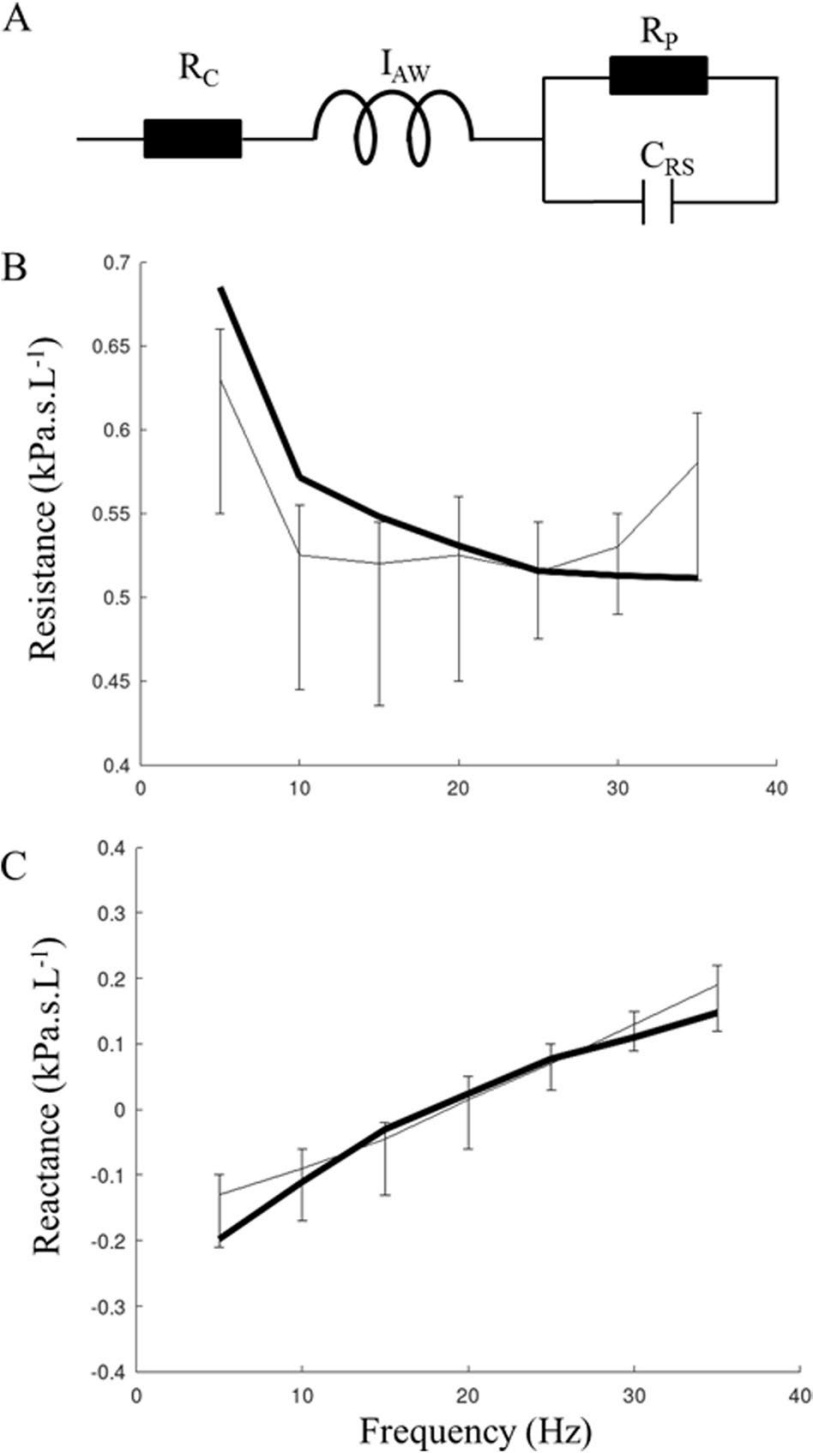
The eRIC model (**A**) and the impedance spectra obtained by fitting the model. Respiratory system resistance (**B**) and reactance (**C**) obtained by fitting the model (thick solid lines) to the data of the experimental measurements represented by the median values and the 25th and 75th percentiles for each frequency.

We used the extended Resistance-Inertance-Compliance model (eRIC) capable of accounting for significant frequency dependence of the respiratory impedance, which has previously been described^16,17^. In eRIC (Fig. 1A), R is partitioned into central (Rc) and peripheral (Rp) resistance of the respiratory system, while C_RS_ is the compliance of the respiratory system (including parenchymal and conducting airways compliances). The model was fitted to the impedance data (5–35 Hz) and the minimization of a performance index allowed the calculation of model parameters, as previously done^17^. We used the corrected Akaike information criterion to evaluate the goodness of fit of the eRIC model on this particular data set^16^.

In all subjects we measured functional residual capacity (FRC) by gas dilution according to American Thoracic Society guidelines^18^. Z scores for FRC were computed with Cook and Herman reference equations for children^19^. Z-scores of IOS variables were calculated according to Gochicoa-Rangel et al.^20^.

### ENT examination

All patients were assessed by an otolaryngologist with endoscopic nasal examination as standard care for moderate to severe OSAS clinical evaluation. Fiber endoscopy was performed with a flexible endoscope after local anesthesia using lidocaine hydrochloride 10%. Nasal obstruction was defined as obstruction from large adenoids (grade 3 or 4 according to Cassano et al.^21^) or turbinate hypertrophy. Oropharyngeal obstruction was defined as presence of obstructive tonsils (grade 3 or 4 according to Brodsky et al.^22^). The ENT specialist was blinded for the results of rhinometry.

### Acoustic rhinometry

The measurements were conducted with the EccoVision Acoustic Rhinometer (E. Benson Hood Laboratories, Pembroke, MA) in the supine position after the subjects were in this position for 5 min, as previously described^23^. The volume of the nasopharynx was recorded and was corrected for height to obtain a normalized parameter. The calculated nasal resistance given by the apparatus (from nostril to nasopharynx) was also recorded. Nasal airway resistance was determined for each side of the nose and the total resistance was calculated using Ohm’s law equation for parallel resistors: 1/R_T_ = 1/R_r_ + 1/R_l_, where R_T_ is the total nasal resistance, R_r_ = nasal resistance on the right side, and R_l_ = nasal resistance on the left side.

### Statistical analysis

We decided to conduct a multivariate analysis with at most five factors, one factor per dimension explored. These factors included two anthropometric factors, namely height and the body mass index (BMI) z-score; one factor from the ENT examination; one factor from the lung function tests; and one factor from the polysomnography data. To perform a multivariate analysis with at most five factors, the sample size of OSAS cases would have to be approximately 50 subjects (10 subjects per factor).

Results were expressed as medians [25–75th percentiles]. Comparisons of continuous variables between groups were performed using the Wilcoxon test or the Kruskal–Wallis test when appropriate. Subsequent intergroup comparisons were performed using the Dunn test for multiple comparisons and *p*-values adjusted with the Benjamini–Hochberg method. Categorical variables were compared using the chi-square test or the Fisher exact test when appropriate. Correlations were evaluated using Pearson's correlation coefficient. Additional statistical analyses are described in the text. A *P* value of less than 0.05 was deemed significant. No correction for multiple testing was done due to the pathophysiological design of the study^24^. All statistical analyses were performed with R software version 4.1.0^25^.

## Results

Sixty-nine children were included in this study all having an AHI ≥ 5/h. From them, 12 children did not produce exploitable tidal breathing recordings because of either insufficient cooperation or leaks/irregular breathing pattern during the recording. The population of excluded children was not different from the remaining population (Supplementary Table S1 online). Two additional children were excluded because of poor quality of the end-tidal CO_2_ record (Supplementary Results online).

**Figure 2:**
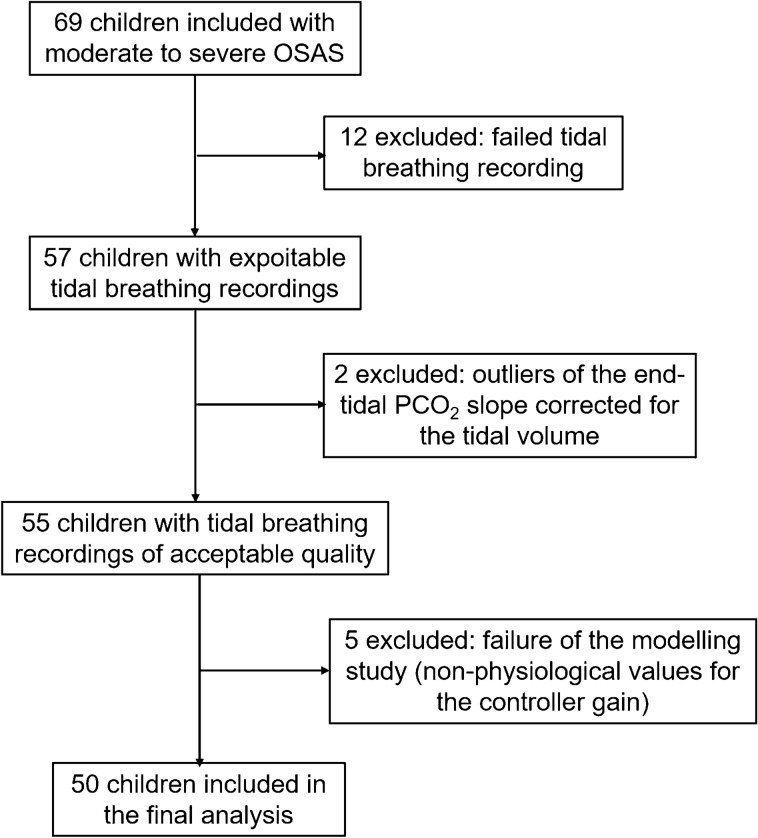
Patient flow chart.

Five additional children were removed from the data set because the analytical model of plant/controller gain produced non-physiological (negative) values for the CG0, their clinical characteristics were similar to the characteristics of the children included in the final analysis (Supplementary Table S2 online). The flow chart of the included children is summarized in Fig. 2. Thus, 50 children were included in the final analysis; their description is given in Table 1. Tables S3 and S4 in Supplementary Results (available online) show the measurements obtained from the tidal breathing analysis and the results of the pulmonary function tests and eRIC modeling respectively. Eleven had physician-diagnosed asthma and their lung function did not differ from non-asthmatic subjects (Table S4 in SupplementaryResults online). The ENT examination found nasal obstruction in 20 children, which was related to the presence of obstructive adenoids (n = 12) and/or to turbinate hypertrophy (n = 10). Twenty-two children had oropharyngeal obstruction corresponding to Brodsky grade 3 or 4. Respiratory system resistance and reactance obtained by fitting the eRIC model to the data of the experimental measurements are shown in Figs. 1B,C. The performance index and the corrected Akaike information criterion were 1.7 [0.9; 2.4] and -78 [− 86; − 73] respectively.

**Table 1:** Clinical characteristics, control of ventilation and lung function parameters in the whole sample and according to the presence of nasal obstruction.

Characteristics	Whole sample N = 50	With nasal obstruction N = 20	Without nasal obstruction N = 30	*P* value	Adjusted* P value
Sex, female/male	17/33	8/12	9/21	0.549	
Age, years	11.2 [7.9; 13.4]	7.1 [5.6; 9.9]	12.6 [10.7; 14.1]	< 0.001	NA
Height, cm	153 [129; 165]	125 [117; 151]	162 [151; 167]	< 0.001	0.254
Weight, kg	64 [30; 99]	29 [21; 56]	86 [62; 107]	< 0.001	0.187
Ethnicity, C/B/A/M	22/20/4/4	9/9/2/0	13/11/2/4	0.474	
Z-score of BMI	2.02 [1.14; 2.57]	1.24 [0.37; 2.00]	2.41 [1.87; 2.63]	0.001	0.553
Neck circumference, cm	34.4 [28.0; 40.0]	28.0 [25.7; 33.7]	38 [33.5; 40.0]	< 0.001	0.112
Asthma, n	11	4	7	> 0.999	
Oropharyngeal obstruction, n	22	11	11	0.323	
**Sleep study data**					
AHI/hour	9.7 [7.5; 20.5]	10.6 [7.4; 18.0]	9.6 [7.6; 20.5]	0.905	0.926
OAHI/hour	8.8 [6.7; 19.2]	9.6 [6.5; 17.5]	8.8 [7.2; 19.2]	0.620	0.851
ODI/hour	8.8 [5.6; 14.0]	8.7 [4.9; 17.2]	9.1 [6.5; 13.6]	0.753	0.899
**Acoustic rhinometry**					
Corrected naso-pharyngeal volume, cm^2^	0.34 [0.27; 0.46]	0.29 [0.24; 0.34]	0.39 [0.31; 0.54]	0.017	0.007
Calculated nasal resistance, cmH_2_O.min.L^− 1^	2.73 [1.80; 4.15]	4.13 [3.01; 6.85]	2.28 [1.80; 3.10]	< 0.001	0.006
**Control of ventilation**					
PG0, mmHg.s.L^− 1^	0.8 [0.6; 1.2]	0.9 [0.6; 1.3]	0.8 [0.5; 1.1]	0.393	0.840
Taup, s	5.0 [3.5; 9.0]	3.9 [2.9; 5.3]	6.3 [4.3; 10.5]	0.025	0.702
GG0, L.s^− 1^.mmHg^− 1^	1.0 [0.4; 3.2]	0.7 [0.2; 3.2]	1.0 [0.6; 3.2]	0.275	0.628
Tauc, s	20.3 [5.1; 50.8]	18.1 [3.9; 51.0]	20.9 [6.2; 50.5]	0.852	0.752
LG0	0.7 [0.2; 2.5]	0.6 [0.2; 2]	1.0 [0.3; 2.5]	0.563	0.583
Delay, s	6.2 [3.7; 8.8]	5.5 [3.4; 9.2]	6.3 [3.8; 8.1]	0.614	0.935
MF-PG, mmHg.s.L^− 1^	0.44 [0.36; 0.57]	0.54 [0.44; 0.75]	0.42 [0.33; 0.51]	0.006	0.732
MF-CG, L.s^− 1^.mmHg^− 1^	0.19 [0.11; 0.29]	0.15 [0.09; 0.23]	0.25 [0.13; 0.33]	0.064	0.760
MF-LG	0.09 [0.05; 0.13]	0.08 [0.06; 0.12]	0.10 [0.05; 0.13]	0.603	0.758
**Lung function**					
Rc, , kPa.s.L^− 1^	0.51 [0.44; 0.63]	0.63 [0.50; 0.91]	0.48 [0.41; 0.54]	0.002	0.710
I, Pa.s^2^.L^− 1^	1.10 [0.89; 1.19]	1.06 [0.88; 1.14]	1.13 [0.93; 1.21]	0.500	0.099
C_RS_, mL.kPa^− 1^	73 [45; 129]	48 [39; 81]	82 [63; 146]	0.037	0.255
Rp, kPa.s.L^− 1^	0.78 [0.49; 1.03]	0.83 [0.64; 1.03]	0.64 [0.46; 1.05]	0.367	0.600

Ethnicities are Caucasian/African/Asian/Mixed.

*P* value stands for the comparison of groups with and without nasal obstruction.

*Statistical tests were adjusted for the presence of oropharyngeal obstruction and age for ventilatory control parameters and only for age for all other parameters (adjusted p value).

### Effect of nasal obstruction on ventilatory control

Clinical, lung function and ventilatory control parameters of the children presenting nasal obstruction and those without are given in Table 1. As age and height were significantly different between the two groups (children with nasal obstruction were younger), we performed statistical analysis adjusted for age as an independent variable. No significant differences in ventilator control parameters were observed between the two groups (Table 1).

### Effect of oropharyngeal obstruction on ventilatory control

**Figure 3:**
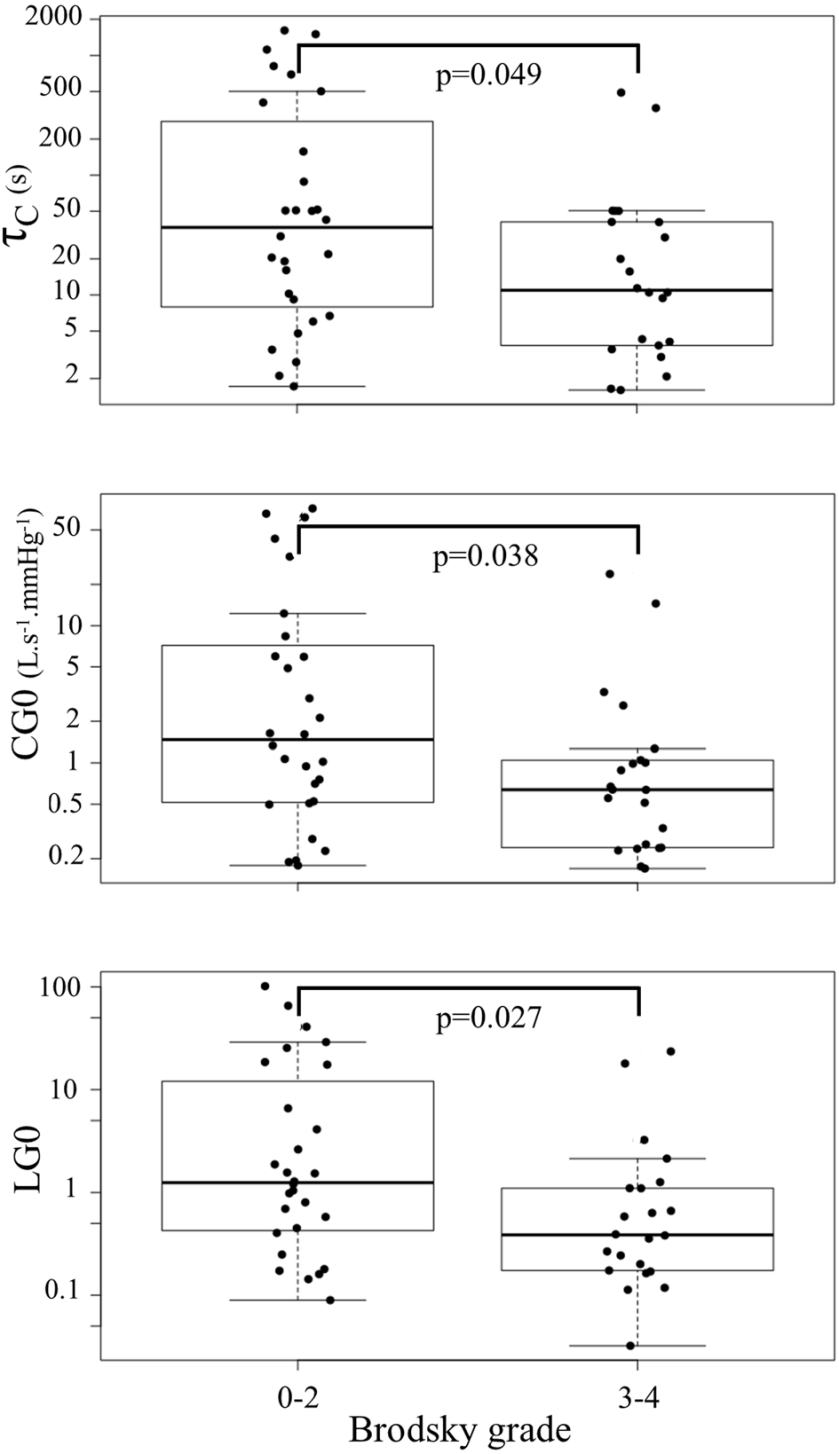
Controller time constant (τ_c_), steady-state controller gain (CG0) and steady-state loop gain (LG0) in children with Brodsky grade 0 to 2 versus children with Brodsky grade 3 to 4 corresponding to oropharyngeal obstruction.

Clinical, lung function and ventilatory control parameters of the children presenting oropharyngeal obstruction and those without are given in Table 2. We found that steady-state CG, controller characteristic time constant and stead-state LG were significantly lower in OSAS children with oropharyngeal obstruction (Table 2 and Fig. 3). Increased central resistance (Rc) was evidenced in children with oropharyngeal obstruction (*p *= 0.028). The analysis of the entire sample of children that had successful ENT examinations and IOS measurements (67 children) further showed that Rc in children with oropharyngeal obstruction was increased compared to those without: 0.56 kPa.s.L^− 1^ [0.49; 0.72] vs 0.48 [0.41; 0.61], respectively; *p *= 0.014. This difference remained significant after adjustment for height (p_adj_ = 0.045).

**Table 2:** Clinical characteristics, control of ventilation and lung function parameters according to the presence of oropharyngeal obstruction.

Characteristics	With oropharyngeal obstruction N = 22	Without oropharyngeal obstruction N = 28	*P* value
Sex, female/male	11/11	6/22	0.042
Age, years	10.4 [6.1; 12.8]	12.1 [9.0; 13.6]	0.184
Height, cm	151 [123; 162]	157 [142; 167]	0.107
Weight, kg	60 [27; 98]	70 [44; 97]	0.353
Ethnicity, C/B/A/M	11/8/3/0	11/12/1/4	0.174
Z-score of BMI	2.00 [1.33; 2.59]	2.21 [1.08; 2.56]	0.807
Neck circumference, cm	33.0 [27.0; 37.0]	35.8 [29.8; 40.0]	0.154
Asthma, n	4	7	0.734
Nasal obstruction, n	11	9	0.323
**Sleep study data**			
AHI/hour	12.1 [7.6; 23.9]	9.4 [7.5; 15.0]	0.384
OAHI/hour	12.0 [7.0; 22.3]	8.3 [6.5; 14.0]	0.237
ODI/hour	9.8 [5.3; 15.5]	8.0 [6.0; 11.7]	0.345
**Acoustic rhinometry**			
Corrected naso-pharyngeal volume, cm^2^	0.36 [0.29; 0.49]	0.33 [0.27; 0.44]	0.468
Calculated nasal resistance, cmH_2_O.min.L^− 1^	2.31 [1.69; 4.14]	2.92 [2.11; 4.14]	0.511
**Control of ventilation**			
PG0, mmHg.s.L^− 1^	0.7 [0.6; 1.2]	0.9 [0.6; 1.2]	0.635
Taup, s	4.0 [2.8; 7.9]	6.0 [4.3; 9.2]	0.103
GG0, L.s^− 1^.mmHg^− 1^	0.6 [0.2; 1.0]	1.5 [0.5; 6.6]	0.038
Tauc, s	10.9 [3.9; 40.6]	36.6 [8.5; 219.1]	0.049
LG0	0.4 [0.2; 1.1]	1.2 [0.4; 9.3]	0.027
Delay, s	6.2 [3.4; 9.5]	6.1 [3.8; 8.2]	0.868
MF-PG, mmHg.s.L^− 1^	0.49 [0.36; 0.71]	0.43 [0.37; 0.54]	0.432
MF-CG, L.s^− 1^.mmHg^− 1^	0.17 [0.11; 0.26]	0.21 [0.12; 0.32]	0.764
MF-LG	0.08 [0.05; 0.11]	0.11 [0.06; 0.14]	0.504
**Lung function**			
Rc, kPa.s.L^− 1^	0.55 [0.49; 0.81]	0.47 [0.38; 0.60]	0.028
I, Pa.s^2^.L^− 1^	0.97 [0.86; 1.15]	1.12 [1.02; 1.22]	0.115
C_RS_, mL.kPa^− 1^	51 [44; 145]	74 [56; 119]	0.789
Rp, kPa.s.L^− 1^	0.94 [0.60; 1.08]	0.66 [0.48; 1.00]	0.274

Ethnicities are Caucasian/African/Asian/Mixed.

P value stands for the comparison of groups with and without oropharyngeal obstruction.

### Relationship between plant gain and lung function

The correlations found between PG and lung function described by the eRIC model are given in Table 3. We found that steady-state PG decreased with increasing minute ventilation (${\dot{\mathrm{V}}}_{\mathrm{e}}$) and BMI z-score in both univariate (r = − 0.55, *p *< 0.001 and r = − 0.54, *p *< 0.001 respectively) and multivariate analyses (adjusted r^2^ = 0.36, *p *< 0.001).

**Table 3:** Plant correlations. Models are given in the first column and the corresponding model coefficients (unstandardized beta) estimates [95% CI] are shown in the line corresponding to a given model.

Model	${\dot{\mathrm{V}}}_{\mathrm{e}}$	Z score BMI	FRC	C_RS_	African ethnicity
PG0 ~ Ve	− 0.09 [− 0.13; − 0.05] *P *< 0.001				
PG0 ~ Z score BMI		− 0.21 [− 0.30; − 0.12] *P *< 0.001			
PG0 ~ Ve + Z score BMI	− 0.06 [− 0.11; − 0.02] *P *= 0.008	− 0.13 [− 0.24; − 0.03] *P *= 0.013			
Taup ~ FRC			3.1 [1.3; 4.9] *P *= 0.001		
Taup ~ C_RS_				37 [19; 55] *P *< 0.001	
Taup ~ FRC + C_RS_ + African ethnicity			1.4 [− 0.9; 3.7] *P *= 0.233	27 [4; 50] *P *= 0.026	− 2.8 [− 5.3; − 0.3] *P *= 0.033
MF PG ~ Ve + Z score BMI + C_RS_ + African ethnicity	− 0.05 [− 0.06; − 0.02] *P *= 0.001	− 0.04 [0.01; − 0.09] *P *= 0.061		− 0.85 [− 1.62; − 0.08] *P *= 0.038	0.06 [− 0.04; 0.16] *P *= 0.265

Univariate analyses demonstrated that the plant characteristic time constant (τ_p_) was related to FRC (r = 0.44, *p *= 0.001) and to the compliance of the respiratory system, C_RS_ (r = 0.50, *p *< 10^–3^). African ethnicity was associated with faster plant time constants 3.6 s [2.7; 6.1] vs 7.5 s [4.4; 11.9] than in Caucasians, *p *= 0.04 (Kurskal-Wallis test), p_adj_ = 0.03. A multiple regression analysis with τ_p_ as the dependent variable and FRC, C_RS_ and ethnicity as independent variables showed that only C_RS_ (*p *= 0.026) and African ethnicity (*p *= 0.033) remained independent predictors of the plant characteristic time constant (adjusted r^2^ = 0.33, *p *< 0.001) in Table 3.

Medium-Frequency (MF)-PG correlated to minute ventilation (${\dot{\mathrm{V}}}_{\mathrm{e}}$, r = − 0.67, *p *< 0.001), to C_RS_ (r = − 0.41, *p *= 0.004) and BMI z-score (r = − 0.47, *p *< 0.001) but not to AHI. A multiple regression analysis with MF-PG as the dependent variable and ${\dot{\mathrm{V}}}_{\mathrm{e}}$, C_RS_, BMI z-score and ethnicity showed that only ${\dot{\mathrm{V}}}_{\mathrm{e}}$ (*p *= 0.001) and C_RS_ (*p *= 0.038) remained independent predictors of MF-PG (adjusted r^2^ = 0.50, *p *< 0.001) in Table 3.

### Asthma related plant gain modifications

Eleven subjects had physician-diagnosed asthma. It has been shown that in asthmatic subjects PG is related to percent predicted forced expiratory volume in first second (FEV_1_%) and to the FEV_1_/FVC ratio^12^. In asthmatic subjects, respiratory system compliance will be mainly influenced by bronchial muscle tone, and hence, the relationship between PG and C_RS_ may be less significant.

**Figure 4:**
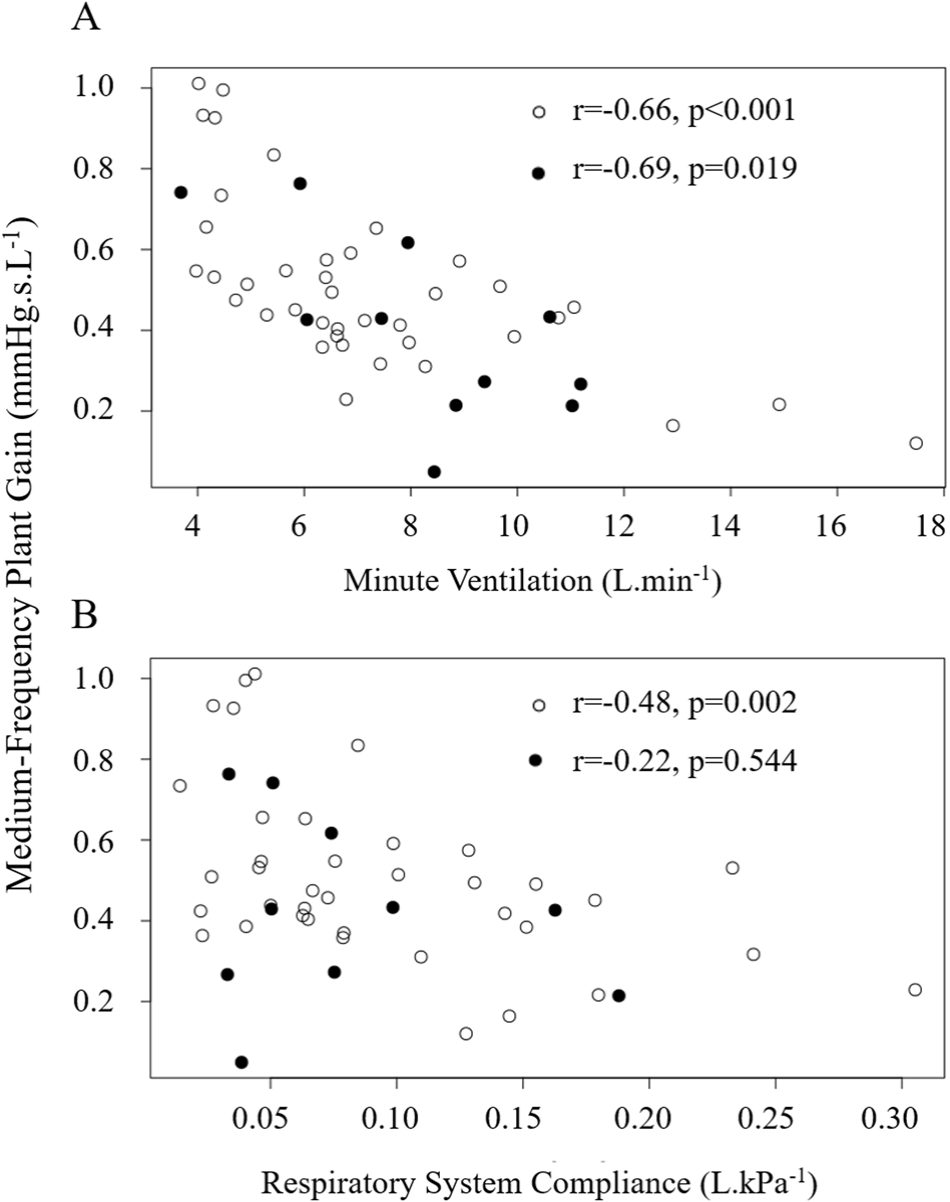
Medium-Frequency Plant Gain (MF-PG) as a function of minute ventilation ${\dot{\text{V}}}_{e}$ (**A**) and the compliance of the respiratory system (C_RS_) (**B**) in non-asthmatics (circles) and in asthmatics (solid circles) with moderate to severe OSAS.

We found that, MF-PG was no longer related to C_RS_ in asthmatic subjects (Fig. 4B), while the relation between MF-PG and ${\dot{\mathrm{V}}}_{\mathrm{e}}$ remained significant (Fig. 4A). In non-asthmatic subjects, we found that MF-PG correlated to obstructive AHI (r = 0.36, *p *= 0.023) and to all previously described parameters (${\dot{\mathrm{V}}}_{\mathrm{e}}$, r = − 0.66, *p *< 0.001, BMI z-score r = − 0.42, *p *= 0.008, C_RS_ r = − 0.48, *p *= 0.002) in univariate analysis.

A multiple regression analysis in the subgroup of non-asthmatic subjects with MF-PG as the dependent variable and ${\dot{\mathrm{V}}}_{\mathrm{e}}$, C_RS_, BMI z-score and obstructive AHI showed that only ${\dot{\mathrm{V}}}_{\mathrm{e}}$ (*p *= 0.001) and C_RS_ (*p *= 0.024) remained independent predictors of MF-PG in this subgroup (adjusted r^2^ = 0.52, *p *< 0.001).

## Discussion

The most significant results of this pathophysiological study in children suffering from moderate to severe OSAS is the observation that oropharyngeal obstruction is associated with decreased steady-state CG and LG and that PG is related to pulmonary function (negative correlation with ventilation and compliance of the respiratory system).

The relationship between oropharyngeal obstruction and ventilatory control is corroborated by previous studies in children with sleep disordered breathing that reported a decrease in LG in children presenting oropharyngeal obstruction^26^. Here, we advance one step further in explaining this decrease by the decrease in CG. One may hypothesize that the decrease in CG is an adaptive response to the increase in the resistance of proximal airways. We evidenced an increase in Rc, corresponding to the resistance of the central airways in the eRIC model of the respiratory system in the group of children with oropharyngeal obstruction. Steady-state CG is the ratio of the increase in ventilatory drive to the increase in arterial CO_2_, thus a reduced controller gain would be more advantageous from a work of breathing perspective. The decrease in controller characteristic time constant is less intuitive to explain, but τ_c_ corresponds to the time during which an increase in PaCO_2_ will stimulate the ventilatory drive. Its modification observed in children with OSAS and with oropharyngeal obstruction could also be interpreted as an adaptive response to the increased proximal airway resistance. The fact that the steady-state CG and LG, and not only the MF-CG or MF-LG, are decreased by oropharyngeal obstruction means that the amplitude of the response of the ventilator drive after a stepwise increase in CO_2_ (or decrease in ventilation for loop gain) would be reduced. We did not evidence any relationship between ventilatory stability parameters and nasal obstruction. This could be explained by the obligatory mouth breathing route during sleep in subjects with nasal obstruction, shunting the nasopharynx. Thus, one may hypothesize that children with nasal obstruction may have similar modifications of ventilatory control than children with pharyngeal obstruction if these modifications are related to the route of breathing. We show that these modifications are related to the presence of oropharyngeal obstruction (Table 2), whatever the presence of nasal obstruction. Nasal obstruction inducing mouth breathing is known to increase the work of breathing in healthy subjects^27^. One may hypothesize that oropharyngeal obstruction will induce more work of breathing than nasal obstruction without oropharyngeal obstruction, leading to the adaptive response of ventilatory control.

We also evidenced that MF-PG decreased with increased minute ventilation and compliance of the respiratory system. The relationship with minute ventilation may seem trivial as ventilation is linked to alveolar PCO_2_ by the equation of alveolar ventilation^28^, but here we found a relationship that applies to different subjects and not to the variations of ventilation for a given subject.

Moreover, it is expected from the model of Khoo and colleagues^29^ (eq. S1 in Supplementary Information online) that steady-state PG (PG0) decreases with increasing alveolar ventilation and shunt fraction. We found that the two independent predictors (negatively related) of PG0 were minute ventilation (${\dot{\mathrm{V}}}_{\mathrm{e}})$ and BMI z-score. The decrease in PG0 with BMI is surprising as some authors have suggested that the reduction of lung volumes due to obesity would produce an increase in PG^7^. In a previous study from our group, we found that MF-PG was decreased in obese control women compared to lean controls although this difference was not significant (0.042 mmHg.min.L^− 1^ [0.022; 0.070] in obese controls vs 0.076 mmHg.min.L^− 1^ [0.031; 0.107] in lean controls)^30^. On the other hand, it is well-known that obesity is associated with increased shunt fraction^31^ that would reduce PG. Our data suggests that obesity decreases PG by two mechanisms: via the increase in both ${\dot{\mathrm{V}}}_{\mathrm{e}}$ ^32^ and pulmonary shunt.

Concerning the plant characteristic time constant (τ_P_), it is expected from eq. S2 (in Supplementary Information online) that τ_P_ scales as alveolar volume and is decreased by ventilation inhomogeneity. We found that τ_P_ increased with lung volume (FRC) and decreased with decreasing C_RS_, which was expected as C_RS_ decreases with increasing ventilation inhomogeneities^33^. We also found that African ethnicity was associated with decreased τ_P_, which could be explained by reduced pulmonary volume compared to Caucasian ethnicity^34^.

When investigating factors related to MF-PG, we found that only minute ventilation and respiratory system compliance remained independent predictors of MF-PG. Thus, the positive correlation between PG and AHI reported by two groups^5,6^ is possibly explained by the up-regulation of inflammatory pathways known to contribute to airway inflammation in patients with OSAS^35,36^. OSAS is a disorder associated with oxidative stress, up-regulation of redox-sensitive genes and inflammatory cascade^35^. OSAS is associated with increased prevalence of neutrophilic pattern in the sputum, thus modifying airway inflammation^37^. These well described pathways support the hypothesis that the inflammatory response associated with OSAS could lead to small airway inflammation and remodeling, which would reduce the compliance of the respiratory system. This relationship is lost in asthmatic patients with OSAS whose bronchial inflammation relies on other inflammatory pathways, which is associated with changes in airway remodelling^38^, while C_RS_ reflects mainly the stiffness of the conducting airways and the elastance of the alveolar tissue explained by the negligible contribution of the chest wall to total respiratory system impedance in children and in the frequency range of IOS, i.e. 5-35Hz^39^. Thus, the normalization of plant gain after adenotonsillectomy observed by Armoni Domany and colleagues^6^ could be explained by the resolution of OSAS and consequent down-regulation of inflammatory pathways leading to improved peripheral lung function.

Our findings also havepotential clinical implications. We confirmed the decrease in LG in children with oropharyngeal obstruction and moderate to severe OSAS. Thus, a more stable chemical control of ventilation is expected in these children arguing against the participation of this endotypic trait in OSAS severity. Therefore, they would be less likely to respond to treatments as oxygen that reduces AHI in adult patients treated for OSAS presenting elevated LG^40^. LG has been identified as a predictor of treatment success of oral appliance therapy along with upper airway collapsibility in adults with OSAS^41^. Namely, a greater response to therapy occurred in those patients with a mild anatomic compromise and a lower LG. Oral appliances are also used to treat OSAS in children, and it has been shown to reduce the AHI by 60% in a small randomized controlled study^42^. Lower LG is expected in children with OSAS and oropharyngeal obstruction, increasing the chances of success of oral appliances. In this case, oral appliances could be used as a treatment option for those children not eligible for adenotonsillectomy, because of surgical contraindication or refusal of the procedure, which remains a first line therapy in children with OSAS and adenotonsillar hypertrophy^10^. PG was increased in children with impaired lower airway mechanics but not specifically in asthmatics, advocating in favor of an effect of OSAS per se on lung mechanics. Thus, the decrease in PG observed after adenotonsillectomy is probably secondary to the improvement of OSAS rather than a proper PG-related effect. The identification of children with residual OSAS and increased PG after adenotonsillectomy should permit clinicians to spot an endotype that could respond to targeted treatment as acetazolamide. Acetazolamide is a carbonic anhydrase inhibitor that produces metabolic acidosis, yielding an increase in baseline ventilation and hence reduces PG. Edwards et al.^43^ observed a 50% reduction in AHI after treatment with acetazolamide in adults with moderate to severe OSAS while the PG was reduced by 40%.

Our study has strengths. First, we used tidal breathing records during wakefulness to evaluate ventilatory control, which is the recommended technique to measure chemical loop gain^44^. Indeed, to evaluate chemical LG in OSAS patients, it is necessary to measure it when the upper airway is stable, for example by measuring ventilatory responses during wakefulness or while the patient is asleep on continuous positive airway pressure (CPAP). When patients with OSAS sleep, there is prevalent snoring and flow limitation that usually continue during the open phase, following an apnea, thus loop gain measurements from polysomnography-driven signals are possibly hampered by the upper airway muscles responsiveness. In snoring children, the upper airway is more stable than in snoring adults^45^ thus, loop gain measurements made during sleep (off CPAP) are still probably acceptable and could be compared to our results. Second, we present a unique dataset from children suffering from moderate to severe OSAS combining upper airway and lower airway assessments.

Our cross-sectional study has limitations. Only children suffering from moderate to severe OSAS were included. First, it was related to the observational design of our study; since in our center, the extensive medical check-up including nasal endoscopy is only carried out in children with a formal therapeutic indication (AHI ≥ 5/hour, moderate to severe OSAS) in accordance with European recommendations^10^. Second, we previously showed that PG explained only 10% of AHI variance; thus in children suffering from moderate to severe OSAS, both normal and elevated values of PG would be observed, allowing the examination of correlations with lung function indices.

In conclusion, we showed that controller and loop gains are reduced in children evaluated for moderate to severe OSAS and presenting oropharyngeal obstruction while nasal obstruction was not associated with a modification of ventilatory control parameters. Controller and loop gains are probably reduced in this population in response to the increased resistance of proximal/central airways. Impairment of lower airways by reducing the compliance of the respiratory system was associated with increased plant gain. This study provides a better understanding of the changes in ventilatory control associated with OSAS in children.

## Supplementary Information

Supplementary Information.

## Data Availability

The data that support the findings of this study are available from the corresponding author upon request. The data are not publicly available because they contain information that may compromise the privacy of the research participants.
