# Supplementary material for: Oropharyngeal obstruction and respiratory system compliance are linked to ventilatory control parameters in pediatric obstructive sleep apnea syndrome
Source: Sci Rep. 2022 Oct 15;12:17340. doi: 10.1038/s41598-022-22236-7 (PMC9569362; doi:10.1038/s41598-022-22236-7)

Supplementary Information

to

Oropharyngeal obstruction and respiratory system compliance are linked to ventilatory control parameters in pediatric obstructive sleep apnea syndrome

Plamen Bokov[1*], Imene Boujemla[2], Boris Matrot[3], Karen Spruyt[3], Jorge Gallego[3] and Christophe Delclaux[1]

[1]: Université de Paris-Cité, AP-HP, Hôpital Robert Debré, Service de Physiologie Pédiatrique-Centre du Sommeil, INSERM NeuroDiderot, F-75019 Paris, France

[2]: AP-HP, Hôpital Robert Debré, Service d'Oto-Rhino-Laryngologie, F-75019 Paris, France

[3]: INSERM NeuroDiderot, F-75019 Paris, France

SUPPLEMENTARY METHODS

End-expiratory CO_2 slope

The method for the calculation of the expiratory slope was adapted from Ream et al.[1] We first defined the end-expiratory phase (phase III of capnography) over a fixed range of fraction of CO_2 in expired gas versus expired volume plot. The midpoint of this phase was defined at the alveolar volume (V_A), where 55% of the expired carbon dioxide had been exhaled with the margins established at volume points ± 20%V_A. Alveolar volume was computed as tidal volume minus the estimate of anatomical dead space. The latter volume corresponded to the minimal volume considered acceptable for tidal volume based on analysis of the subject's tidal breathing record. The raw slopes of individual breaths (expressed as $mmHg.L^{-1}$) were normalized by the mean value of CO_2 expiratory pressure obtained over the entire expiratory cycle[1] and, thus, expressed as L^{-1}. This normalization allowed comparison of breaths with differing rates of CO_2 excretion.

Age has been shown to be a major determinant of the expiratory slop[1]; hence, the slope was further normalized by multiplying it by the mean tidal volume of the subject. This procedure permitted us to search for outliers corresponding to subjects that could have poorly performed during the tidal breathing recording.

We analyzed suspected outliers using the interquartile range (IQR) criterion. The IQR criterion states that all observations above q0.75+1.5·IQR or below q0.25−1.5·IQR (where q0.25 and q0.75 correspond to first and third quartile respectively, and IQR is the difference between the third and first quartile) are considered to be potential outliers.

SUPPLEMENTARY RESULTS

Sixty-nine children were included in this study all having an AHI ≥ 5/h. From these, twelve children did not produce exploitable tidal breathing recordings because of either insufficient cooperation or leaks/irregular breathing pattern during the recording. The comparison of the population of excluded children with the remaining population is given in Table S1. Figure S1A presents the relationship between the slope of expiratory PCO_2 and age in the remaining 57 children. The analysis of the slope of expiratory PCO_2 multiplied by the tidal volume ($S*V_T$) permitted us to identify two outliers (asterisk), using the IQR criterion (Figure S1B). The expiratory slope negatively correlated with age ($r = -0.56$, $p < 0.001$). The relationship was no longer significant when the slope was multiplied by the tidal volume (Figure S1B, $r = -0.03$, $p = 0.830$). These two recordings were excluded from further analysis.

Five additional children were removed from the data set because the analytical model of plant/controller gain produced non-physiological (negative) values for the CG0; their clinical characteristics are summarized in Table S2.

Tables S3 and S4 show the measurements obtained from the tidal breathing analysis and the results of the pulmonary function tests and eRIC modeling, respectively. Eleven had physician-diagnosed asthma; their lung function is compared to the lung function parameters of the non-asthmatics in Table S4.

SUPPLEMENTARY DISCUSSION

We propose to add to the discussion the model presented by Khoo et al.[2] It deals with a model of plant gain integrating ventilation-perfusion mismatch. According to this model, steady-state plant gain (PG0) and characteristic plant time constant could be written as follows:

$$(S1) \qquad PG0 = \frac{(1 - F_S)P_{ACO_2}}{\acute{V}_A + 863Qk(1 - F_s)}$$

$$(S2) \qquad \tau_P = \frac{(1 - F_v)V_L}{\acute{V}_A + 863Qk(1 - F_s)}$$

In Eqn.S2, a fraction Fv of the total lung volume V_L and a fraction Fs of total perfusion Q are assumed to not participate in the gas exchange process. P_{ACO2} represent alveolar CO_2 partial pressure and $\acute{V}_A$ is the alveolar ventilation, while k is the slope of the blood CO_2 dissociation curve. The pair (Fv = 0, Fs = 0) corresponds to the case of the homogeneously ventilated and fully perfused lung (i.e., no ventilation-perfusion mismatch). Other values 0 < Fv and Fs < 1 correspond to various degrees of ventilation-perfusion mismatch.

According to the model, medium-frequency plant gain is the mean of the plant transfer function, which relates ΔP_{ACO_2} to $\Delta\acute{V}_A$ over the frequency range of periodic breathing, i.e.

$$(S3) \qquad MF - PG = \left| \frac{PG0}{\sqrt{(2\pi f\tau_P)^2 + 1}} \right|_{f=\frac{1}{15}}^{f=\frac{1}{5}}$$

Thus, the model predicts a decrease of MF-PG with increasing shunt or alveolar ventilation. On the other hand, as Fv increases, the corresponding reduction in effective gas exchange volume reduces the time constant, τ_P, for CO_2 washout in the lungs, which in turn increases MF-PG.

References

1. Ream, R. S. *et al.* Volumetric Capnography in Children Influence of Growth on the Alveolar Plateau Slope. *Anesthesiol. J. Am. Soc. Anesthesiol.* **82**, 64–73 (1995).
2. Khoo, M. C. K., Hu, W.-H. & Amin, R. Effects of Ventilation-Perfusion Mismatch on Severity of Obstructive Sleep Apnea: A Modeling Study. in *2020 42nd Annual International Conference of the IEEE Engineering in Medicine Biology Society (EMBC)* 2792–2795 (2020). doi:10.1109/EMBC44109.2020.9175297.

Table S1. Clinical characteristics and control of ventilation parameters in the children that performed acceptable tidal breathing recording versus those that did not.

Characteristics	Accepted recordings N=57	Disregarded N=12	P value
Sex, female/male	21/36	4/8	1
Age, years	11.1 [7.7; 13.4]	10.8 [6.3; 12.0]	0.401
Height, cm	152 [128; 164]	151 [120; 161]	0.652
Weight, kg	66 [30; 100]	61 [22; 83]	0.433
Ethnicity, C/B/A/M	25/24/4/4	8/2/1/1	0.284
Z-score of BMI	2.15 [1.11; 2.60]	1.95 [0.09; 2.31]	0.346
Neck circumference, cm	34.4 [28.0; 40.0]	31.0 [27.3; 36.3]	0.376
**Sleep study data**			
AHI/hour	9.7 [7.5; 20.3]	12.4 [8.3; 23.8]	0.624
OAHI/hour	8.8 [6.8; 18.4]	13.7 [7.4; 24.5]	0.434
ODI/hour	8.7 [5.3; 14.5]	8.1 [5.5; 19.3]	0.703
**Lung Function**			
Rc, $kPa.s.L^{-1}$	0.52 [0.45; 0.62]	0.53 [0.47; 0.66]	0.738
I, $Pa.s^2.L^{-1}$	1.1 [0.9; 1.2]	1.0 [0.8; 1.4]	0.800
C_{RS}, $mL.kPa^{-1}$	64 [45; 119]	56 [38; 112]	0.701
Rp, $kPa.s.L^{-1}$	0.77 [0.52; 1.02]	0.67 [0.44; 0.94]	0.284
**Acoustic rhinometry**			
Corrected naso-pharyngeal volume, cm^2	0.34 [0.26; 0.45]	0.38 [0.31; 0.44]	0.585
Calculated nasal resistance, $cmH_2O.min.L^{-1}$	2.91 [1.91; 4.46]	3.88 [2.10; 6.44]	0.394
**ENT examination**			
Nasal Obstruction, No/Yes	32/24	5/7	0.358
Oropharyngeal Obstruction, No/Yes	33/24	6/6	0.751

Ethnicities are Caucasian/African/Asian/Mixed.

Table S2. Clinical characteristics and control of ventilation parameters in the children with physiologically acceptable controller gain model values and those in which the model did not produce physiologically acceptable values.

Characteristics	Accepted N=50	Disregarded N=5	P value
Sex, female/male	17/33	2/3	1
Age, years	11.2 [7.9; 13.4]	11.8 [5.4; 13.4]	0.918
Height, cm	153 [129; 165]	158 [114; 161]	0.578
Weight, kg	65 [30; 99]	99 [17; 131]	0.759
Ethnicity, C/B/A/M	22/20/4/4	2/3/0/0	0.853
Z-score of BMI	2.02 [1.14; 1.62]	2.57 [-0.30; 2.63]	0.861
Neck circumference, cm	34.4 [28.0; 40.0]	37.0 [25.0; 41.0]	0.917
**Sleep study data**			
AHI/hour	9.7 [7.5; 20.5]	7.8 [7.8; 11.6]	0.682
OAHI/hour	8.8 [6.7; 19.2]	7.5 [6.8; 11.4]	0.770
ODI/hour	8.8 [5.6; 14.0]	6.5 [2.4; 20.0]	0.633
**Lung Function**			
Rc, $kPa.s.L^{-1}$	0.51 [0.44; 0.63]	0.54 [0.51; 0.62]	0.712
I, $Pa.s^2.L^{-1}$	1.1 [0.9; 1.2]	1.2 [1.1; 1.3]	0.318
C_{RS}, $mL.kPa^{-1}$	73 [45; 129]	56 [52; 60]	0.153
Rp, $kPa.s.L^{-1}$	0.78 [0.49; 1.03]	0.72 [0.63; 0.80]	0.941
**Acoustic rhinometry**			
Corrected naso-pharyngeal volume, cm^2	0.34 [0.27; 0.46]	0.21 [0.17; 0.28]	0.076
Calculated nasal resistance, $cmH_2O.min.L^{-1}$	2.73 [1.81; 4.14]	5.83 [3.54; 9.41]	0.064
**ENT examination**			
Nasal Obstruction, No/Yes	29/20	2/3	0.640
Oropharyngeal Obstruction, No/Yes	28/22	4/1	0.387
**Tidal breathing quality parameters**			
End-tidal PCO2 slope , L-1	4.0 [2.5; 6.3]	3.6 [3.6; 6.1]	0.759
Slope*Tidal Volume	1.53 [1.10; 2.00]	1.80 [1.71; 2.36]	0.183

Ethnicities are Caucasian/African/Asian/Mixed.

Table S3. Tidal breathing analysis parameters

Characteristics, median [25th ; 75th percentile]	50 children
Minute ventilation, L/min	6.8 [5.5; 8.8]
$PetCO_2$, mmHg	37.6 [35.9; 38.7]
$PetO_2$, mmHg	104.2 [99.0; 108.4]
End-tidal PCO_2 slope , L^{-1}	4.0 [2.5; 6.3]
Tidal volume, L	0.40 [0.27; 0.54]
Slope*Tidal Volume	1.53 [1.10; 2.00]

Table S4. Lung function test parameters in asthmatic and non-asthmatic children

Characteristics, median [25th ; 75th percentile]	Non-asthmatic children N=39	Asthmatic children N=11	P value
Age	10.5 [7.2; 12.9]	13.0 [12.1; 13.8]	0.073
**IOS indices**			
R20Hz, Z-score	1.90 [1.37; 2.89]	2.15 [1.35; 3.01]	0.832
R5Hz, Z-score	1.76 [0.70; 2.39]	2.27 [1.66; 4.09]	0.194
R5-20Hz, Z-score	0.67 [-0.17; 1.38]	1.57 [0.67; 2.71]	0.082
Fres, Z-score	0.57 [-0.65; 1.15]	1.22 [-0.54; 1.79]	0.387
X5Hz, Z-score	-0.46 [-1.08; 0.09]	-1.35 [-1.95; -0.22]	0.139
Ax, Z-score	0.89 [0.23; 1.59]	2.14 [1.13; 2.65]	0.065
**eRIC model parameters**			
Rc, $kPa.s.L^{-1}$	0.52 [0.44; 0.65]	0.48 [0.46; 0.55]	0.662
I_{AW}, $Pa.s^2.L^{-1}$	1.1 [0.9; 1.2]	1.1 [1.1; 1.2]	0.387
C_{RS}, $mL.kPa^{-1}$	74 [46; 130]	63 [41; 93]	0.644
Rp, $kPa.s.L^{-1}$	0.80 [0.50; 1.02]	0.69 [0.51; 1.13]	0.950
FRC, z-score	-1.0 [-1.8; 0.1]	-1.6 [-2.5; -1.0]	0.101

P value stands for the comparison of groups with and without asthma.

SUPPLEMENTARY FIGURE LEGEND

Figure S1. Slope of the expiratory CO_2 as a function of age (in A) and the corrected slope by the mean tidal volume as a function on age (in B). Two outliers in the corrected slope are identified by asterisk, seen also in A. The corrected slope is independent of age while the slope was related to age. All correlations were done after exclusion of the two outliers.

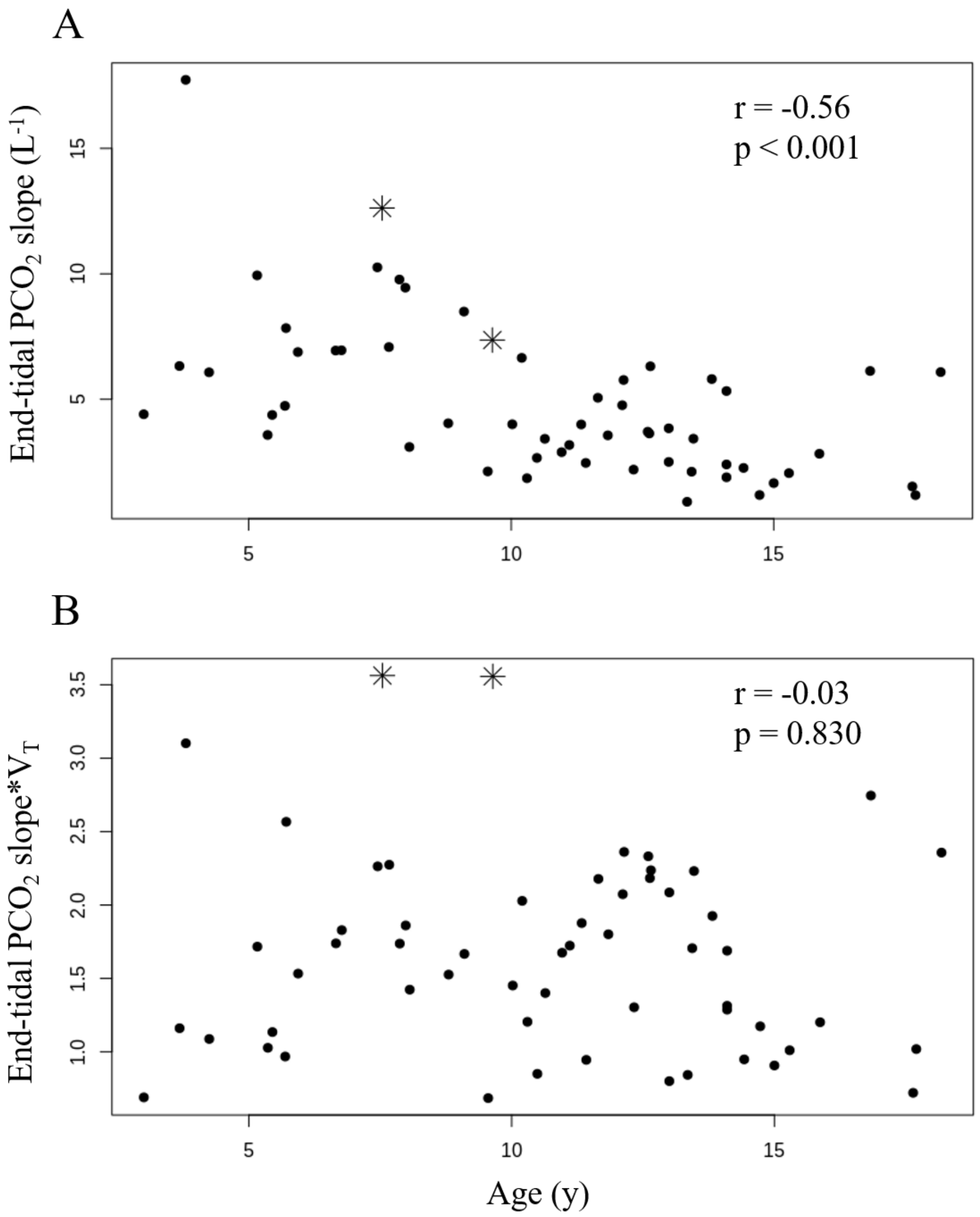

Supplement: Supplementary file 1 — Supplementary Information. [file 41598_2022_22236_MOESM1_ESM.pdf]
